# Prognostic Models for Predicting Overall Survival in Patients with Primary Gastric Cancer: A Systematic Review

**DOI:** 10.1155/2019/5634598

**Published:** 2019-09-18

**Authors:** Qi Feng, Margaret T. May, Suzanne Ingle, Ming Lu, Zuyao Yang, Jinling Tang

**Affiliations:** ^1^Division of Epidemiology, JC School of Public Health and Primary Care, The Chinese University of Hong Kong, Hong Kong, China; ^2^Population Health Sciences, Bristol Medical School, University of Bristol, Bristol, UK; ^3^Key Laboratory of Carcinogenesis and Translational Research (Ministry of Education), Department of GI Medical Oncology, Peking University Cancer Hospital & Institute, Beijing, China; ^4^Cochrane Hong Kong, JC School of Public Health and Primary Care, The Chinese University of Hong Kong, Hong Kong, China; ^5^Shenzhen Municipal Key Laboratory for Health Risk Analysis, Shenzhen Research Institute of the Chinese University of Hong Kong, Shenzhen, China

## Abstract

**Background:**

This study was designed to review the methodology and reporting of gastric cancer prognostic models and identify potential problems in model development.

**Methods:**

This systematic review was conducted following the CHARMS checklist. MEDLINE and EMBASE were searched. Information on patient characteristics, methodological details, and models' performance was extracted. Descriptive statistics was used to summarize the methodological and reporting quality.

**Results:**

In total, 101 model developments and 32 external validations were included. The median (range) of training sample size, number of death, and number of final predictors were 360 (29 to 15320), 193 (14 to 9560), and 5 (2 to 53), respectively. Ninety-one models were developed from routine clinical data. Statistical assumptions were reported to be checked in only nine models. Most model developments (94/101) used complete-case analysis. Discrimination and calibration were not reported in 33 and 55 models, respectively. The majority of models (81/101) have never been externally validated. None of the models have been evaluated regarding clinical impact.

**Conclusions:**

Many prognostic models have been developed, but their usefulness in clinical practice remains uncertain due to methodological shortcomings, insufficient reporting, and lack of external validation and impact studies.

**Impact:**

Future research should improve methodological and reporting quality and emphasize more on external validation and impact assessment.

## 1. Introduction

Although gastric cancer has been decreasing in terms of both incidence and mortality in most developed countries in recent decades, it still causes substantial disease burden [1]. Over 90% of people with early stage gastric cancer survive for five years or longer after surgical treatment [2, 3], whereas the prognosis of those with advanced gastric cancer is poor, with a 5-year survival rate of about 20% in stage III and 7% in stage IV patients, respectively [4].

Risk stratification is important in informing treatment decision, resource allocation, and patient recruitment in clinical trials [5]. The tumor-node-metastasis (TNM) staging system is widely used for risk stratification [4]. Nevertheless, even patients with the same TNM stage may have significantly different responses to treatment and clinical outcomes [6], suggesting that more accurate stratification could be beneficial [7]. Previous studies have identified numerous other prognostic factors for gastric cancer, which can be broadly divided into four categories: patient-, tumor status-, biomarker-, and treatment-related factors [8–10]. As no single prognostic factor suffices for satisfactory risk stratification, much interest has been raised in developing multivariable prognostic models, which quantitatively combine two or more prognostic factors [11, 12].

The American Joint Committee on Cancer has increasingly recognized the importance of incorporating prognostic models into practice to achieve personalized cancer management [13]. However, despite plenty of prognostic models published in the literature, very few have been adopted in clinical use. We carried out this systematic review to summarize the characteristics of existing models for predicting overall survival of patients with primary gastric cancer, with an emphasis on identifying potential problems in model development and validation and informing future research.

## 2. Methods

This systematic review was conducted following the *CHARMS checklist* [14], which was developed to guide data extraction and critical appraisal in systematic reviews of prediction model studies.

### 2.1. Eligibility Criteria

We included primary studies that reported the development and/or validation of prognostic models predicting overall survival of patients with primary gastric cancer. A prognostic model was defined as a combination of at least two prognostic factors, based on multivariable analysis, to estimate individual risk of a specific outcome, presented as regression formula, nomogram, or in a simplified form, such as risk score [15–17]. We only included prognostic models for predicting overall survival or all-cause death, excluding other outcomes, such as progression-free survival after treatment or disease-specific survival.

We excluded studies if (1) they enrolled patients with other types of cancer and the information on gastric cancer model could not be separately extracted; (2) they used short-term mortality (for example, death within 30 days after surgery) as the outcome; or (3) they validated prognostic models that were not initially developed for gastric cancer patients.

### 2.2. Literature Search

We searched MEDLINE and EMBASE to identify all relevant studies from their inceptions through 30 May 2018, using the following three groups of terms: (1) *gastric tumor^∗^* OR *gastric tumour^∗^* OR *gastric cancer^∗^* OR *gastric neoplasm^∗^* OR *gastric carcinoma^∗^* OR *stomach tumor^∗^* OR *stomach tumour^∗^* OR *stomach cancer^∗^* OR *stomach neoplasm^∗^* OR *stomach carcinoma^∗^* OR *Siewert* OR *esophagogastric* OR *EGJ*, and (2) *prognos^∗^* OR *survival* OR *death* OR *mortality*, and (3) *scor^∗^* OR *model^∗^* OR *index^∗^* OR *nomogram^∗^* OR *rule^∗^* OR *predict^∗^* OR *indices* OR *formula^∗^* OR *equation^∗^* OR *algorithm*^∗^ [18, 19]. The search terms were limited to title and abstract. We also manually checked the reference lists of eligible studies to identify extra relevant studies.

### 2.3. Study Selection

After excluding duplicates, we screened all titles and abstracts to identify potentially eligible studies and then retrieved their full texts for further examination. Final eligibility was confirmed by two authors (QF and ZYY). Discrepancy was resolved by discussion with a third author (JLT).

### 2.4. Data Extraction and Quality Assessment

The data extraction form was designed according to the *CHARMS checklist* [14], supplemented with other items obtained from methodological guidance studies and previous systematic reviews [15, 16]. Briefly, the following information was extracted for each model development and external validation: publication year, country, data source, patient characteristics, length of follow-up, outcome, candidate predictors, training sample size, number of deaths, missing data, model development/validation methods, final predictors, predictive performance, and presentation formats.

In this study, candidate predictor refers to the potential predictors (and their functional forms, if any) that are selected to be examined in multivariable analysis but might or might not be included in final model. Final predictor refers to the predictors that are included in final models. Event per variable (EPV) is the ratio between the number of events and the number of candidate predictors, which is a rule of thumb to empirically evaluate the power of regression analysis, with a value of 10 or higher recommended to avoid potential overfitting [20, 21]. If one study included multiple model developments and validations, we extracted relevant information for each model development and validation separately. Data extraction was undertaken by two authors (QF and ZYY), and any uncertainty in data extraction was resolved by discussion with a third author (JLT).

We used a preliminary version of the *Prediction Model Study Risk Of Bias Assessment Tool* (PROBAST) to evaluate the methodological quality of each model development and validation [22]. This tool evaluated the levels of risk of bias in five domains: participant selection, definition and measurement of predictors, definition and measurement of outcome, sample size and participant flow, and statistical analysis. Each domain was rated as high, low, or unclear risk of bias. Overall judgment of risk of bias was derived from the judgments on all domains: low risk if all domains had low risk of bias, high risk if any domain had high risk of bias, otherwise unclear risk.

### 2.5. Statistical Analysis

We mainly used descriptive statistics to summarize the characteristics of model developments and validations. All final predictors were assigned into one of the four categories: patient, tumor status, biomarker, and treatment. We counted the frequency of each final predictor being included in models. We compared models that have been externally validated with those that have not, regarding their characteristics (training sample size, number of events, number of final predictors, EPV, c statistic, etc.).

## 3. Results

**Figure 1:**
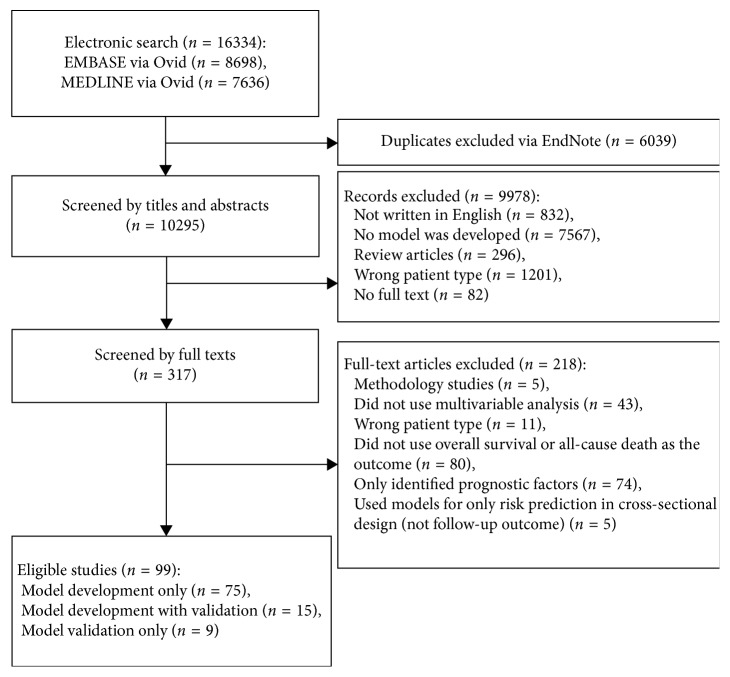
The flowchart of study selection.

In total, 16334 citations were identified and 99 eligible publications (Supplementary [Supplementary-material supplementary-material-1]) were included in this review (Figure 1). Of the 99 publications, 75 performed model development only, 9 performed external validation only, and 15 performed both model development and external validation. One-hundred and one distinct models were extracted from 90 studies. Thirty-two external validations for 20 models were extracted from 24 studies.

### 3.1. Model Development

#### 3.1.1. Basic Characteristics of Model Development

**Figure 2:**
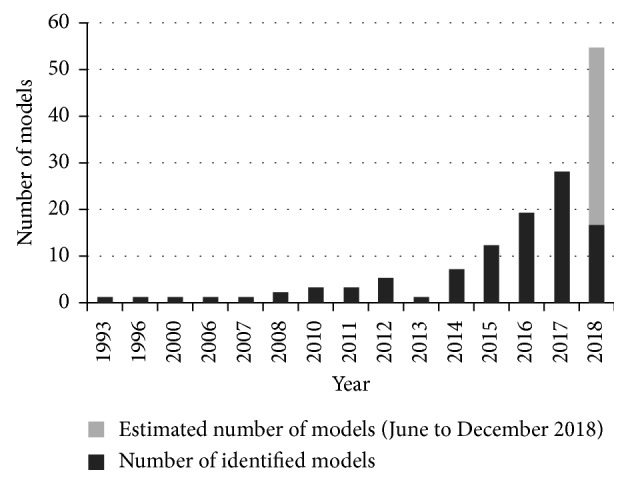
Number of published prognostic models by publication year. The estimated number of prognostic model in 2018 was calculated based on the assumption that the model number was proportionate to the number of months. We found 16 models through 30^th^ May in 2018, and the estimated model number in 2018 would be 16 *∗*(12/5)=38.4.

Characteristics of the 101 model developments are summarized in Table 1. Three models were published before 2000, and the number has rapidly risen by 2- and 30-fold during 2001–2010 and 2011–2018, respectively (Figure 2). Most models (76/101) originated from East Asian populations, which was not surprising, given the fact that this region has the highest incidence and prevalence of gastric cancer.

Patient characteristics varied substantially across studies in terms of age, sex, tumor status, and treatment (Table 1). The median proportion of male patients was 67.8% (range 30.9% to 80.3%), and the median age was 60 years (range 51 to 70). Thirty-six models were developed from gastric cancer patients at TNM stages I–III only and 17 models from TNM stage IV only. Most models (71/101) recruited only patients who had received surgery.

#### 3.1.2. Summary of Model Development Methods

Most models (91/101) were developed by retrospective cohort studies based on routine clinical data, which were not collected for the purpose of model development. To deal with missing data, which is a common problem with routine clinical data, seven models adopted the multiple imputation approach, while the remaining 94 models conducted complete-case analysis. The medians of total sample size and number of events included in analysis were 360 (range 29 to 15320) and 193 (range 14 to 9560), respectively. The starting point of follow-up for overall survival varied across models. Seven studies did not report their candidate predictors clearly. EPV can be estimated in 83 model developments, with the median of 25.1 (range 0.2 to 1481.3). A favored EPV (>10) was achieved in 64 model developments.

As for selection of candidate predictors, 63 models used univariable analysis, 30 models prespecified candidate predictors based on clinical knowledge, five models employed a combination of the two, and the other three models did not specify this issue clearly. Various statistical models were used for prognostic model development, with Cox proportional hazard model being the most popular one (used in 90 models). Sixty-eight models used a stepwise approach in multivariable analyses to select final predictors. Statistical assumptions of the methods were examined and reported in only nine studies.

The median number of final predictors was 5 (range 2 to 10). In total, 180 different predictors were included, of which 21 were patient-related, 34 tumor-related, 116 biomarkers, and 9 treatment-related (Table 2; more details in the Supplementary [Supplementary-material supplementary-material-1]). The most consistent predictors for overall survival (included by more than 10 models) were age at diagnosis, sex, lymph node involvement, metastasis, invasion depth, TNM stage, tumor size, tumor site, differentiation, and histologic type, all of which were patient- and tumor-related.

The models were mostly presented in simplified forms, such as risk score (35/101) and nomogram (47/101). For model performance, 33 and 55 models did not report discrimination and calibration, respectively. Among the studies reporting relevant information, the median c statistic for discrimination was 0.748 (range 0.627 to 0.961). Forty-two models were compared with TNM stage alone regarding c statistic value, and all models outperformed TNM stage, with a median increase in c statistic value of 0.050 (range 0.015 to 0.180).

### 3.2. External Validation

There were 32 external validations for 20 distinct models, with 22 of them reporting in the same study as the model development. The majority (81/101) of models developed have not been externally validated. Five models were externally validated more than once, and two models [23, 24] more than five times. The characteristics of training datasets and validation datasets were compared in 19 external validations. Five validations did not assess discrimination, and 24 did not assess calibration (Table 3). The median (range) of c statistic for discrimination was 0.770 (0.576 to 0.868). The difference in c statistic between development and validation ranged from −0.044 to 0.290 with a median of 0.029.

### 3.3. Quality Assessment

The model developments had either high (97/101) or unclear (4/101) risk of bias, and all model validations had high risk of bias. Ninety-one developments and 31 validations had high risk of bias in participant selection, mainly due to retrospective data collection. Forty-six developments and six validations had high risk of bias in sample size and participant flow, mainly due to small sample size and inappropriate method of dealing with missing data. Eighty-three developments and 13 validations had high risk of bias in analysis, mainly due to inappropriate method dealing with continuous variable, lack of statistical assumption examination, lack of overfitting detection, and insufficient reporting of model performance (Supplementary [Supplementary-material supplementary-material-1]).

### 3.4. Comparison of Externally Validated Models with Not-Validated Models

When comparing development characteristics of externally validated models with not-validated models, we found that the validated models tended to have larger training sample size, bigger number of events, higher EPV, older age, and higher c statistic value, while the differences in number of final predictors seemed to be insignificant (Table 4). Multivariable logistic regression showed that models were more likely to be externally validated if they were developed with bigger training sample and higher c statistic.

## 4. Discussion

This systematic review identified 101 models predicting overall survival of gastric cancer patients, with 20 of them externally validated.

van den Boorn et al. published a systematic review [25] summarizing prediction models for esophageal and/or gastric cancer patients, but the present study substantially differed from it and has its own value. Firstly, this study focused on prognostic models designed for primary gastric cancer patients only, whereas van den Boorn et al. included models for both gastric and esophageal cancers. Secondly, we identified 40 more newly published models and provided a more comprehensive picture of their characteristics. Thirdly, van den Boorn et al. focused on models' performance and clinical application, but our study emphasized more on the methodology of model development and validation.

We observed substantial heterogeneity regarding patient types in model development. Many studies developed prognostic models for specific subgroups of gastric cancer patients (e.g., those with a certain tumor stage and those receiving certain treatment) to make their models unique from those developed by others. This strategy of patient restriction may limit the model's generalizability, increasing uncertainty when applying it to other types of patients. In addition, an underlying assumption of restricting a model development to specific patient subgroups is that there exists effect modification or interaction between the restriction variable(s) and the main prognostic factors of interest. However, most studies did not check this assumption.

We also identified common statistical shortcomings that may cause bias in model development. Firstly, most models were developed from routinely collected clinical data, in which missing data was common. Most models simply performed complete-case analysis by excluding the patients with missing data. However, complete-case analysis works well only when missing data occurs completely at random, which is rare in reality [26]. To address this issue, multiple imputation has been recommended [27, 28]. This method has been employed in prediction model studies of other diseases [29, 30] but has not been applied in gastric cancer until 2017 [31–33].

Secondly, univariable analysis was commonly used to select candidate predictors. However, this data-driven method has high risk of wrongly excluding a potentially significant variable or including a potentially insignificant variable when its association with the outcome is confounded by others [34, 35]. The bootstrap resampling method could be used to increase the stability of variable selection, by selecting variables with high inclusion frequency across multiple bootstrapping samples [36]. Moreover, variable selection should take into account clinical or biological knowledge and combine results of multivariable analysis with sensitivity analysis for cautious conclusion [34, 35].

Thirdly, the majority of studies did not examine the assumptions of the statistical models, such as hazard-proportionality for Cox regression and linearity assumption for continuous variables. The results of examination are important in selecting appropriate statistical models and determining predictors' functional forms [37, 38]. Cox regression is the most commonly used model for survival data, but the underlying proportional-hazard assumption was often violated, in which case parametric survival models could be considered [39, 40]. In addition, algorithms from machine learning (e.g., neural network) are less strict with assumptions and have been used more and more in prognostic model development.

Fourthly, detection of model overfitting was neglected in most studies. Overfitting is more likely to occur in studies with small sample size and many predictors, resulting in overestimation of risk in high-risk patients and underestimation low-risk ones [41]. This issue can be detected by internal validation with cross-validation or bootstrap resampling and handled with statistical methods, such as shrinkage and penalized regression [42, 43].

Underreporting is another common problem. Outcome definitions, variable selection method, assessment of discrimination, and calibration measures were not reported in 34%, 23%, 33%, and 55% of model developments, respectively. Because there is no standard method for model development and multiple feasible options exist at each step in model development, underreporting of methodological details may cause difficulty in assessing a model's internal and external validity. Future studies are suggested to follow relevant reporting guidelines such as the *TRIPOD* [44, 45].

In this study, we found 55 predictors that were included more than twice in models, 10 of which (age at diagnosis, sex, lymph node involvement, metastasis, invasion depth, TNM stage, tumor size, tumor site, differentiation, and histologic type) were included more than 10 times. This can be regarded as indirect evidence for their predictive power in gastric cancer prognosis. Direct evidence, i.e., magnitude of their association with the outcome, such as hazard ratio, can be found in previous systematic reviews and meta-analyses [46, 47]. Therefore, we suggest that future model development, if necessary, to build upon these existing evidence. Though a large number of biomarkers were studied, their frequency of being included in final models was very low (mostly once or twice).

Prognostic models can be used to inform patients of their prognosis and assist clinical decision-making. However, despite the much effort devoted into model development so far, very few prediction models other than the TNM stage system have been adopted in clinical practice. Apart from the problems discussed above, other reasons may include the complexity of those models as compared with TNM system and lack of external validation and clinical impact studies [48]. External validation evaluates a model's predictive performance in local setting and updates model if necessary. An impact study evaluates the effects of a prognostic model on clinical decision-making, behavioral change, subsequent health outcomes of individuals, and the cost-effectiveness of applying the model, with the optimal design being a clustered randomized controlled trial [49, 50]. The gap between prognostic model and clinical decision rule is another big concern. Prognostic models compute the probability of an event on a continuous scale or risk scores on an ordinal scale, whereas clinical decision is a binary choice regarding whether to use an intervention or not. Unfortunately, the translation of risk estimates derived from existing prognostic models to clinical decisions is much less investigated [50].

Therefore, future research should try to avoid repeatedly developing new models for similar predictive purposes with small sample size and high risk of bias. Instead, more emphasis should be put on improving methodological quality of model development, validating and updating models for use within their own setting [51], translating model prediction into clinical decision rules [50], and assessing the models' clinical impact [52, 53].

## 5. Conclusion

This systematic review identified 101 prognostic models for predicting overall survival of patients with gastric cancer, which were limited by high risk of bias, methodological shortcomings, insufficient reporting, and lack of external validation and clinical impact assessment. Future prognostic model research should pay more attention to their methodological and reporting quality, and more importantly, emphasized more on external validation and impact studies to assess the models' effectiveness in improving clinical outcomes.

## Figures and Tables

**Table 1:** Characteristics of 101 model developments.

	Model developments (*n* = 101)
*Study characteristics*	
Publication year	
Before 2000	3
2001–2010	7
2011–2018	91
Study location	
East Asia (China/Japan/Korea)	76
Non-Asian	25
Data source	
Clinical data/retrospective cohort	91
Prospective cohort	7
Randomized controlled trial	3
*Patient characteristics*	
Male% (4/101 missing)	67.6 (30.9, 80.3)^a^
Age (5/101 missing)	
Median (min, max) of mean	60.0 (51.0, 70.0)^a^
Tumor TNM stage	
All	46
I–III	36
IV	17
No information	2
Gastrectomy	
No restriction	28
Only patients with gastrectomy	71
Only patients without gastrectomy	2
*Model development*	
Sample size (training set) (14/101 missing)	360 (29, 15320)^a^
Number of events	193 (14, 9560)^a^
Event per variable (18/101 missing)	25.1 (0.2, 1481.3)^a^
Length of follow-up (month) (53/101 missing)	44.0 (6.7, 111.6)^a^
Start of outcome follow-up	
From diagnosis	3
From surgery	49
From other time points^b^	15
Unclear	34
Candidate selection methods	
Prespecification	30
Univariable analysis	63
Prespecification + univariable analysis	5
Unclear	3
Statistical model	
Cox proportional hazard regression	90
Others^c^	11
Final predictor selection	
Full model	10
Stepwise (including forward and backward)	68
Unclear	23
Statistical assumptions ever checked	9
Number of final predictors	5 (2, 53)^a^
Formats of presentations	
Score	35
Nomogram	47
Equation	9
Others (decision tree and neural network)	4
No	6
Predictive performance	
Discrimination	
AUC/c statistic	67
Others	1
No	33
Calibration	
Calibration plot	45
Hosmer–Lemeshow test	3
No	55
Model validation	
Internal	30
External	21
No	54

^a^Median (min, max). ^b^Initiation of chemotherapy (*n* = 10), metastasis (*n* = 3), and randomization (*n* = 2). ^c^CART, Cox Lasso, discrimination analysis, Weibull model, neural network, and logistic model. AUC: area under curve.

**Table 2:** Final predictors included in the models.

Category	Number of predictors	Number of predictors selected multiple times	Predictors selected multiple times^a^
Patient	21	9	Age, sex, ethnicity, performance score, year of diagnosis, family history, smoking, residency, and addiction to opium

Disease status	34	21	T stage, N stage, TNM stage, tumor site, tumor size, differentiation, metastasis, histologic type, Lauren type, LN ratio, lymphovascular invasion, bone metastasis, Borrmann type, liver metastasis, number of metastasis sites, lung metastasis, number of examined LN, metastasis LN, perineural invasion, LODDS, and TTP after chemotherapy

Biomarker	116	19	CEA, NLR, ALP, albumin, bilirubin, CA199, Hb, CES1, IS, LDH, LNR:ART, lymphocyte count, MGAT5, mGPS, NPTM, platelet, sodium, TNFRSF11A, and WBC

Treatment	9	6	Chemotherapy, gastrectomy, lymphedenectomy, resection margin, extent of resection, and radiotherapy

^a^The table lists only the predictors that have been included more than once. LN: lymph node. LODDS: log odds of positive LN. CEA: carcinoembryonic antigen. NLR: neutrophil/lymphocyte ratio. ALP: alkaline phosphatase. Hb: hemoglobin. MGAT5: *β*1, 6-N-acetylglucosaminyltransferase-5. mGPS: modified Glasgow Prognostic Score. CA199: cancer antigen 199. NPTM: number of positive tumor markers (cancer antigen 125, CA199, CEA). WBC: white blood cell. TTP: time to progression.

**Table 3:** Characteristics of model external validations.

	External validations (*n* = 32)
Data source	
Clinical	27
Prospective cohort	3
Randomized controlled trial	2
Validated in	
The original development study	22
Independent study	10
Sample size for validation	610 (71, 26019)^a^
Discrimination	
AUC/c statistic	25
Others	2
No	5
Calibration	
Calibration plot	6
Hosmer–Lemeshow test	2
Calibration in large	1
No	24
Compared validation set with development set	19

^a^Median (min, max). AUC: area under curve.

**Table 4:** Characteristics of models with external validation and those without.

	Externally validated models (*n* = 20) mean (SD)	Not externally validated models (*n* = 81) mean (SD)	*P* value
Training sample size	3902.55 (5777.62)	634.17 (926.30)	0.021
Number of events	2825.12 (4069.04)	344.75 (613.35)	0.028
Number of candidate predictors	75.80 (204.53)	12.83 (28.26)	0.185
EPV	364.21 (542.04)	44.70 (82.97)	0.033
Number of final predictors	6.65 (3.44)	5.94 (6.08)	0.490
Length of follow-up (month)	64.24 (29.65)	43.76 (19.15)	0.122
Age	63.00 (4.99)	59.87 (3.39)	0.034
Male%	64.92 (4.10)	67.29 (6.54)	0.053
c statistic	0.80 (0.06)	0.75 (0.07)	0.042

EPV: event per variable.

## Supplementary Materials

Supplementary Materials Supplementary file 1: full list of included studies. Supplementary file 2: model presentation and the predictors included in final models. Supplementary file 3: quality assessment of model development and validation.
