# Supplementary material for: Prognostic Models for Predicting Overall Survival in Patients with Primary Gastric Cancer: A Systematic Review
Source: Biomed Res Int. 2019 Sep 18;2019:5634598. doi: 10.1155/2019/5634598 (PMC6766665; doi:10.1155/2019/5634598)
Supplement: Supplementary Materials — Supplementary file 1: full list of included studies. Supplementary file 2: model presentation and the predictors included in final models. Supplementary file 3: quality assessment of model development and validation. [file 5634598.f1.pdf]

Supplementary file 1. Full list of included studies

All included studies [1-99] (n=99):

Development studies [1-26, 28-43, 45-49, 51, 53-61, 63-65, 67-78, 80, 81, 83, 84, 86-99] (n=90)

Validation studies (independently-external validation studies, and development studies including external validation) [4, 8, 17, 25, 27, 28, 44, 47, 48, 50, 52, 56, 62, 63, 66, 73, 75, 79, 82, 83, 85, 87, 90, 94] (n=24)

1. Liu H, Shen Z, Wang X*, et al.* Increased expression of C-C motif ligand 2 associates with poor prognosis in patients with gastric cancer after gastrectomy. Tumor Biology 2016;37(3):3285-3293.

2. Wang W, Chen XL, Zhao SY*, et al.* Prognostic significance of preoperative serum CA125, CA19-9 and CEA in gastric carcinoma. Oncotarget 2016;7(23):35423-35436.

3. Rades D, Huttenlocher S, Bartscht T*, et al.* Predicting the survival probability of gastric cancer patients developing metastatic epidural spinal cord compression (MESCC). Gastric Cancer 2015;18(4):881-884.

4. Wang X, He H, Zhang H*, et al.* Clinical and prognostic implications of beta1, 6-N-acetylglucosaminyltransferase V in patients with gastric cancer. Cancer Science 2012;104(2):185-193.

5. Bria E, De Manzoni G, Beghelli S*, et al.* A clinical-biological risk stratification

model for resected gastric cancer: Prognostic impact of HER2, FHIT, and APC expression status. Annals of Oncology 2013;24(3):693-701.

6. Zhu X, Zhao X, Peng W*, et al.* Epirubicin combined with oxaliplatin and 5-day continuous infusion of 5-fluorouracil as a first-line treatment for metastatic gastric cancer: treatment outcomes and analysis of prognostic factors. Journal of Cancer Research and Clinical Oncology 2015;141(1):109-118.

7. Mohri Y, Tanaka K, Ohi M*, et al.* Prognostic significance of host- and tumor-related factors in patients with gastric cancer. World Journal of Surgery 2010;34(2):285-90.

8. Woo Y, Son T, Song K*, et al.* A novel prediction model of prognosis after gastrectomy for gastric carcinoma. Annals of Surgery 2016;264(1):114-120.

9. Victorzon M, Lundin J, Haglund C*, et al.* A risk score for predicting outcome in patients with gastric cancer, based on stage, sialyl-Tn immunoreactivity and ploidy - A multivariate analysis. International Journal of Cancer 1996;67(2):190-193.

10. Costa ML, de Cassia Braga Ribeiro K, Machado MA*, et al.* Prognostic score in gastric cancer: the importance of a conjoint analysis of clinical, pathologic, and therapeutic factors. Annals of Surgical Oncology 2006;13(6):843-50.

11. Haga Y, Ikejiri K, Wada Y*, et al.* Preliminary study of surgical audit for overall survival following gastric cancer resection. Gastric Cancer 2015;18(1):138-146.

12. Catalano V, Graziano F, Santini D*, et al.* Second-line chemotherapy for patients with advanced gastric cancer: Who may benefit? British Journal of Cancer 2008;99(9):1402-1407.

13. Park HS, Rha SY, Kim HS*, et al.* A prognostic model to predict clinical outcome in gastric cancer patients with bone metastasis. Oncology 2011;80(1-2):142-150.

14. Wang Q, Yang Y, Zhang YP*, et al.* Prognostic value of carbohydrate tumor markers and inflammation-based markers in metastatic or recurrent gastric cancer. Medical oncology (Northwood, London, England) 2014;31(12):289.

15. Kanagavel D, Pokataev IA, Fedyanin MY*, et al.* A prognostic model in patients treated for metastatic gastric cancer with second-line chemotherapy. Annals of Oncology 2010;21(9):1779-1785.

16. Wang X, Zhang H, He H*, et al.* Prognostic value of stromal cell-derived factor 1 expression in patients with gastric cancer after surgical resection. Cancer Science 2014;105(11):1447-56.

17. Han DS, Suh YS, Kong SH*, et al.* Nomogram predicting long-term survival after D2 gastrectomy for gastric cancer. Journal of Clinical Oncology 2012;30(31):3834-3840.

18. Song BI, Kim HW, Won KS*, et al.* Preoperative standardized uptake value of metastatic lymph nodes measured by 18F-FDG PET/CT improves the prediction of

prognosis in gastric cancer. Medicine 2015;94(26):e1037.

19. Xu ZY, Chen JS, Shu YQ. Gene expression profile towards the prediction of patient survival of gastric cancer. Biomedicine and Pharmacotherapy 2010;64(2):133-139.

20. Zhang H, Wang X, Shen Z*, et al.* Infiltration of diametrically polarized macrophages predicts overall survival of patients with gastric cancer after surgical resection. Gastric Cancer 2015;18(4):740-750.

21. Wang Z, Liu H, Shen Z*, et al.* The prognostic value of CXC-chemokine receptor 2 (CXCR2) in gastric cancer patients. BMC Cancer 2015;15(1):766.

22. Mohammadzadeh F, Noorkojuri H, Pourhoseingholi MA*, et al.* Predicting the probability of mortality of gastric cancer patients using decision tree. Irish Journal of Medical Science 2015;184(2):277-84.

23. Min L, Ruan Y, Shen Z*, et al.* Overexpression of Ras-GTPase-activating protein SH3 domain-binding protein 1 correlates with poor prognosis in gastric cancer patients. Histopathology 2015;67(5):677-688.

24. Koo DH, Ryoo BY, Kim HJ*, et al.* A prognostic model in patients who receive chemotherapy for metastatic or recurrent gastric cancer: validation and comparison with previous models. Cancer Chemotherapy and Pharmacology 2011;68(4):913-921.

25. Zhang H, Wang X, Xu J*, et al.* Notch1 activation is a poor prognostic factor in

patients with gastric cancer. British Journal of Cancer 2014;110(9):2283-2290.

26. Liu K, Yang K, Wu B*, et al.* Tumor-infiltrating immune cells are associated with prognosis of gastric cancer. Medicine 2015;94(39):e1631.

27. Reim D, Novotny A, Eom BW*, et al.* External validation of an Eastern Asian nomogram for survival prediction after gastric cancer surgery in a European patient cohort. Medicine 2015;94(52):e2406.

28. Eom BW, Ryu KW, Nam BH*, et al.* Survival nomogram for curatively resected Korean gastric cancer patients: Multicenter retrospective analysis with external validation. PLoS ONE 2015;10(2):e0119671.

29. Qian J, Qian Y, Wang J*, et al.* A clinical prognostic scoring system for resectable gastric cancer to predict survival and benefit from paclitaxel- or oxaliplatin-based adjuvant chemotherapy. Drug Design, Development and Therapy 2016;10:241-258.

30. Qu JL, Qu XJ, Li Z*, et al.* Prognostic model based on systemic inflammatory response and clinicopathological factors to predict outcome of patients with node-negative gastric cancer. PLoS ONE 2015;10(6):e0128540.

31. Xu YQ, Jiang TW, Cui YH*, et al.* Prognostic value of ABO blood group in patients with gastric cancer. Journal of Surgical Research 2016;201(1):188-195.

32. Kim JG, Ryoo BY, Park YH*, et al.* Prognostic factors for survival of patients with advanced gastric cancer treated with cisplatin-based chemotherapy. Cancer

Chemotherapy and Pharmacology 2008;61(2):301-307.

33. Kologlu M, Kama NA, Reis E*, et al.* A prognostic score for gastric cancer. American Journal of Surgery 2000;179(6):521-526.

34. Du C, Zhou Y, Huang K*, et al.* Defining a high-risk subgroup of pathological T2N0 gastric cancer by prognostic risk stratification for adjuvant therapy. Journal of Gastrointestinal Surgery 2011;15(12):2153-2158.

35. Becker K, Reim D, Novotny A*, et al.* Proposal for a multifactorial prognostic score that accurately classifies 3 groups of gastric carcinoma patients with different outcomes after neoadjuvant chemotherapy and surgery. Annals of Surgery 2012;256(6):1002-1007.

36. Lee J, Lim T, Uhm JE*, et al.* Prognostic model to predict survival following first-line chemotherapy in patients with metastatic gastric adenocarcinoma. Annals of Oncology 2007;18(5):886-891.

37. Wang J, Qu J, Li Z*, et al.* A prognostic model in metastatic or recurrent gastric cancer patients with good performance status who received first-line chemotherapy. Translational Oncology 2016;9(3):256-261.

38. Lei KF, Liu BY, Zhang XQ*, et al.* Development of a survival prediction model for gastric cancer using serine proteases and their inhibitors. Experimental and Therapeutic Medicine 2012;3(1):109-116.

39. Kadowaki S, Komori A, Narita Y*, et al.* Long-term outcomes and prognostic factors of patients with advanced gastric cancer treated with S-1 plus cisplatin combination chemotherapy as a first-line treatment. International Journal of Clinical Oncology 2014;19(4):656-661.

40. Hirabayashi S, Kosugi S, Isobe Y*, et al.* Development and external validation of a nomogram for overall survival after curative resection in serosa-negative, locally advanced gastric cancer. Annals of Oncology 2014;25(6):1179-84.

41. Takahari D, Boku N, Mizusawa J*, et al.* Determination of prognostic factors in Japanese patients with advanced gastric cancer using the data from a randomized controlled trial, Japan clinical oncology group 9912. The oncologist 2014;19(4):358-366.

42. Liu H, Zhang H, Shen Z*, et al.* Expression of Jagged1 predicts postoperative clinical outcome of patients with gastric cancer. International Journal of Clinical and Experimental Medicine 2015;8(9):14782-14792.

43. Song KY, Park YG, Jeon HM*, et al.* A nomogram for predicting individual survival of patients with gastric cancer who underwent radical surgery with extended lymph node dissection. Gastric Cancer 2014;17(2):287-293.

44. Mariani L, Miceli R, Lusa L*, et al.* A modified prognostic score for patients with curatively resected gastric cancer. Tumori 2005;91(3):221-226.

45. Wang ZX, Yang XL, He MM*, et al.* The Efficacy of adjuvant FOLFOX6 for patients with gastric cancer after D2 lymphadenectomy a propensity score-matched analysis. Medicine (United States) 2016;95(16):e3214.

46. Liu H, Zhang H, Shen Z*, et al.* Increased expression of CSF-1 associates with poor prognosis of patients with gastric cancer undergoing gastrectomy. Medicine (United States) 2016;95(9):e2675.

47. Marubini E, Bonfanti G, Bozzetti F*, et al.* A prognostic score for patients resected for gastric cancer. European Journal of Cancer Part A: General Topics 1993;29(6):845-850.

48. Wang Z, Chen G, Wang Q*, et al.* Identification and validation of a prognostic 9-genes expression signature for gastric cancer. Oncotarget 2017;8(43):73826-73836.

49. Wang P, Wang Y, Hang B*, et al.* A novel gene expression-based prognostic scoring system to predict survival in gastric cancer. Oncotarget 2016;7(34):55343-55351.

50. Akgul O, Ocak S, Gundogdu SB*, et al.* Comparison of East and West survival nomograms in Turkish gastric cancer patients who underwent radical surgery. Scand J Surg 2018; 10.1177/1457496918766724:1457496918766724.

51. Bauer L, Hapfelmeier A, Blank S*, et al.* A novel pretherapeutic gene expression-based risk score for treatment guidance in gastric cancer. Ann Oncol 2018;29(1):127-132.

52. Bencivenga M, Verlato G, Han DS, *et al.* Validation of two prognostic models for recurrence and survival after radical gastrectomy for gastric cancer. Br J Surg 2017;104(9):1235-1243.

53. Cao Y, Liu H, Zhang H, *et al.* CXC chemokine receptor 1 predicts postoperative prognosis and chemotherapeutic benefits for TNM II and III resectable gastric cancer patients. Oncotarget 2017;8(12):20328-20339.

54. Cao Y, Zhang H, Liu H, *et al.* Glycoprotein 130 is associated with adverse postoperative clinical outcomes of patients with late-stage non-metastatic gastric cancer. Sci Rep 2016;6:38364.

55. Chen H, Li H, Zhao J, *et al.* High intratumoral expression of tetranectin associates with poor prognosis of patients with gastric cancer after gastrectomy. J Cancer 2017;8(17):3623-3630.

56. Chen S, Rao H, Liu J, *et al.* Lymph nodes ratio based nomogram predicts survival of resectable gastric cancer regardless of the number of examined lymph nodes. Oncotarget 2017;8(28):45585-45596.

57. Choi JH, Suh YS, Choi Y, *et al.* Comprehensive Analysis of the Neutrophil-to-Lymphocyte Ratio for Preoperative Prognostic Prediction Nomogram in Gastric Cancer. World J Surg 2018; 10.1007/s00268-018-4510-4.

58. De B, Rhome R, Jairam V, *et al.* Gastric adenocarcinoma in young adult patients:

patterns of care and survival in the United States. Gastric Cancer 2018; 10.1007/s10120-018-0826-x.

59. Deng X, Xiao Q, Liu F*, et al.* A gene expression-based risk model reveals prognosis of gastric cancer. PeerJ 2018;6:e4204.

60. Fuchs CS, Muro K, Tomasek J*, et al.* Prognostic factor analysis of overall survival in gastric cancer from two phase III studies of second-line Ramucirumab (REGARD and RAINBOW) using pooled patient data. J Gastric Cancer 2017;17(2):132-144.

61. Goshayeshi L, Hoseini B, Yousefli Z*, et al.* Predictive model for survival in patients with gastric cancer. Electron Physician 2017;9(12):6035-6042.

62. Haga Y, Hato S, Ikenaga M*, et al.* Validation of an assessment tool: estimation of postoperative overall survival for gastric cancer. Eur J Surg Oncol 2018;44(4):515-523.

63. Hou JY, Wang YG, Ma SJ*, et al.* Identification of a prognostic 5-Gene expression signature for gastric cancer. J Cancer Res Clin Oncol 2017;143(4):619-629.

64. Hung YS, Chang SC, Liu KH*, et al.* A prognostic model based on lymph node metastatic ratio for predicting survival outcome in gastric cancer patients with N3b subclassification. Asian J Surg 2017; 10.1016/j.asjsur.2017.10.001.

65. Jiang Y, Zhang Q, Hu Y*, et al.* ImmunoScore signature: a prognostic and predictive tool in gastric cancer. Ann Surg 2018;267(3):504-513.

66. Kim PS, Lee KM, Han DS*, et al.* External validation of a gastric cancer nomogram

derived from a large-volume center using dataset from a medium-volume center. J Gastric Cancer 2017;17(3):204-211.

67. Kim SY, Yoon MJ, Park YI*, et al.* Nomograms predicting survival of patients with unresectable or metastatic gastric cancer who receive combination cytotoxic chemotherapy as first-line treatment. Gastric Cancer 2018;21(3):453-463.

68. Korhani Kangi A, Bahrampour A. Predicting the survival of gastric cancer patients using artificial and Bayesian neural networks. Asian Pac J Cancer Prev 2018;19(2):487-490.

69. Lin C, Liu H, Zhang H*, et al.* Tryptase expression as a prognostic marker in patients with resected gastric cancer. Br J Surg 2017;104(8):1037-1044.

70. Lin C, Liu H, Zhang H*, et al.* Interleukin-13 receptor alpha2 is associated with poor prognosis in patients with gastric cancer after gastrectomy. Oncotarget 2016;7(31):49281-49288.

71. Liu H, Lin C, Shen Z*, et al.* Decreased expression of granulocyte-macrophage colony-stimulating factor is associated with adverse clinical outcome in patients with gastric cancer undergoing gastrectomy. Oncol Lett 2017;14(4):4701-4707.

72. Miao Y, Sui J, Xu SY*, et al.* Comprehensive analysis of a novel four-lncRNA signature as a prognostic biomarker for human gastric cancer. Oncotarget 2017;8(43):75007-75024.

73. Narita Y, Kadowaki S, Oze I*, et al.* Establishment and validation of prognostic nomograms in first-line metastatic gastric cancer patients. J Gastrointest Oncol 2017;9(1):52-63.

74. Peng P, Min L, Song S*, et al.* Elevated expression of Calpain-4 predicts poor prognosis in patients with gastric cancer after gastrectomy. Int J Mol Sci 2016;17(10).

75. Peng PL, Zhou XY, Yi GD*, et al.* Identification of a novel gene pairs signature in the prognosis of gastric cancer. Cancer Med 2018;7(2):344-350.

76. Qu J, Qu X, Li Z*, et al.* Role of patient-, tumor- and systemic inflammatory response-related factors in predicting survival of patients with node-negative gastric cancer. Tumour Biol 2017;39(6):1010428317698374.

77. Song J, Yin J, Bai Z*, et al.* The profile of serum microRNAs predicts prognosis for resected gastric cancer patients receiving platinum-based chemotherapy. Dig Dis Sci 2017;62(5):1223-1234.

78. Sun H, He B, Nie Z*, et al.* A nomogram based on serum bilirubin and albumin levels predicts survival in gastric cancer patients. Oncotarget 2017;8(25):41305-41318.

79. Takahari D, Mizusawa J, Koizumi W*, et al.* Validation of the JCOG prognostic index in advanced gastric cancer using individual patient data from the SPIRITS and G-SOX trials. Gastric Cancer 2017;20(5):757-763.

80. Wang C, Zhou Y, Chen B*, et al.* Prognostic value of tripartite motif containing 29 expression in patients with gastric cancer following surgical resection. Oncol Lett 2018;15(4):5792-5798.

81. Wang PL, Xiao FT, Gong BC*, et al.* A nomogram for predicting overall survival of gastric cancer patients with insufficient lymph nodes examined. J Gastrointest Surg 2017;21(6):947-956.

82. Wang ZX, Li GX, Zhou ZW*, et al.* Validation of a nomogram for selecting patients for chemotherapy after D2 gastrectomy for cancer. Br J Surg 2017;104(9):1226-1234.

83. Wang ZX, Qiu MZ, Jiang YM*, et al.* Comparison of prognostic nomograms based on different nodal staging systems in patients with resected gastric cancer. J Cancer 2017;8(6):950-958.

84. Wen T, Wang Z, Li Y*, et al.* A four-factor immunoscore system that predicts clinical outcome for stage II/III gastric cancer. Cancer Immunol Res 2017;5(7):524-534.

85. Woo Y, Goldner B, Son T*, et al.* Western validation of a novel gastric cancer prognosis prediction model in US gastric cancer patients. J Am Coll Surg 2018;226(3):252-258.

86. Wu S, Liu H, Zhang H*, et al.* Galectin-8 is associated with recurrence and survival of patients with non-metastatic gastric cancer after surgery. Tumour Biol

2016;37(9):12635-12642.

87. Yang J, Bo L, Han T*, et al.* Pathway- and clinical-factor-based risk model predicts the prognosis of patients with gastric cancer. Mol Med Rep 2018;17(5):6345-6356.

88. Yuan SQ, Wu WJ, Qiu MZ*, et al.* Development and validation of a nomogram to predict the benefit of adjuvant radiotherapy for patients with resected gastric cancer. J Cancer 2017;8(17):3498-3505.

89. Zeng D, Zhou R, Yu Y*, et al.* Gene expression profiles for a prognostic immunoscore in gastric cancer. Br J Surg 2018; 10.1002/bjs.10871.

90. Zhang H, Liu H, Shen Z*, et al.* Tumor-infiltrating neutrophils is prognostic and predictive for postoperative adjuvant chemotherapy benefit in patients with gastric cancer. Ann Surg 2018;267(2):311-318.

91. Zhang J, Li SQ, Liao ZH*, et al.* Prognostic value of a novel FPR biomarker in patients with surgical stage II and III gastric cancer. Oncotarget 2017;8(43):75195-75205.

92. Zhao LY, Chen XL, Wang YG*, et al.* A new predictive model combined of tumor size, lymph nodes count and lymphovascular invasion for survival prognosis in patients with lymph node-negative gastric cancer. Oncotarget 2016;7(44):72300-72310.

93. Zheng ZF, Lu J, Zheng CH*, et al.* A novel prognostic scoring system based on

preoperative sarcopenia predicts the long-term outcome for patients after R0 resection for gastric cancer: experiences of a high-volume center. Ann Surg Oncol 2017;24(7):1795-1803.

94. Zhong Q, Chen QY, Li P*, et al.* Prediction of conditional probability of survival after surgery for gastric cancer: a study based on Eastern and Western large data sets. Surgery 2018; 10.1016/j.surg.2018.02.011.

95. Zhou YX, Yang LP, Wang ZX*, et al.* Lymph node staging systems in patients with gastric cancer treated with D2 resection plus adjuvant chemotherapy. J Cancer 2018;9(4):660-666.

96. Oh SE, Seo SW, Choi MG*, et al.* Prediction of overall survival and novel classification of patients with gastric cancer using the survival recurrent network. Ann Surg Oncol 2018;25(5):1153-1159.

97. Coimbra FJF, da Costa WL, Ribeiro HSC*, et al.* Noncurative resection for gastric cancer patients: who could benefit?: Determining prognostic factors for patient selection. Annals of Surgical Oncology 2016;23(4):1212-1219.

98. Hsieh MC, Wang SH, Chuah SK*, et al.* A prognostic model using inflammation-and nutrition-based scores in patients with metastatic gastric adenocarcinoma treated with chemotherapy. Medicine (United States) 2016;95(17):e3504.

99. Liu J, Geng Q, Chen S*, et al.* Nomogram based on systemic inflammatory response markers predicting the survival of patients with resectable gastric cancer after D2 gastrectomy. Oncotarget 2016;7(25):37556-37565.

Supplementary file 2. Model presentation and included final predictors

Model #	Reference #*	Study ID	Model presentation	Included final predictors
1	1	Liu Hao 2016(1)	Nomogram	T, N, M, CCL2 expression
2	2	Wang Wei 2016	Nomogram	Age, tumor size, TNM, NPTM
3	2	Wang Wei 2016	Nomogram	Tumor size, TNM, NPTM, macroscopic type
4	3	Rades Dirk 2015	Unweighted score	Visceral metastasis, rapidity of developing weakness of legs
5	4	Wang Xuefei 2012	No information	TNM, MGAT5 expression
6	4	Wang Xuefei 2012	No information	Kiel stage, MGAT5 expression
7	5	Bria E 2013	HR-weighted score	Sex, age, TNM, resection margin, tumor site, resected LN, Her2, APC
8	6	Zhu Xiaodong 2015	Unweighted score	liver metastasis, lung metastasis, CA199, ascite*pleural effusion
9	7	Mohri Yasuhiko 2010	Coefficient-weighted score	NLR, tumor size, T
10	8	Woo Yanghee 2016	Equation	Age, T, M, number of retrieved LN, number of positive LN, resection type(total/subtotal), sex, histology(differentiation)
11	9	Victorzon M 1996	Coefficient-weighted score	TNM, STn, DNA ploidy

12	10	Costa Marcelo Leite Viera 2006	Coefficient-weighted score	Sex, weight loss, TNM, lymphocyte count, LNR, lymphedenectomy(D)
13	11	Haga Yoshio 2015	Equation	T, N, circumferential involvement, age, ASA physical status, serum sodium
14	12	Catalano V 2008	Unweighted score	ECOG performance status, Hb, CEA, the number of metastasis, TTP after chemotherapy
15	13	Park Hyung Soon 2011	Unweighted score	ECOG performance status, multiple bone metastasis, CEA
16	14	Wang Qing 2014	Unweighted score	CEA, CA125, mGPS
17	15	Kanagavel D 2010	HR-weighted score	ECOG performance score, Hb, TTP under chemotherapy
18	16	Wang Xuefei 2014	Nomogram	T, N, M, SDF-1expression
19	17	Han Dongseok 2012	Nomogram	Age, sex, tumor site, depth of invasion, number of metastatic LN, number of examined LN(square root)
20	18	Song Bongil 2015	No information	T, N, nodal SUVmax
21	19	Xu Zhenyue 2010	Equation (Weights based on z score from univariate)	ITGB1, PDGFB, THBS1, TWIST1
22	20	Zhang Heng 2015	Nomogram	T, N, M, CD11c, CD206
23	21	Wang Zhenglin 2015	Nomogram	T, N, M, CXCR2 expression

24	22	Mohammadzadeh F 2015	Decision tree (with 16 rules)	Diabetes, surgery, tumor size, age, pathologic stage, ethnicity, exposure to chemical exposure, alcohol, tobacco
25	23	Min Lingqiang 2015	No information	T, TNM, G3BP1 expression
26	24	Koo Dong Hoe 2011	HR-weighted score	M, NLR
27	25	Zhang H 2014	Score	TNM, ICN1 expression
28	26	Liu Kai 2015	Nomogram	Tumor site, tumor size, N, resection type(total/subtotal),TFoxp3+ cells, TCD68+/SCD68+ ratio, TCD66b+/SCD66b+ ratio, TCD8+/TFoxp3+ ratio
29	28	Eom Bang Wool 2015	Nomogram	Age, lymphedenectomy, tumor site, lymphovascular invasion, tumor size, depth of invasion, metastatis LN
30	29	Qian Jing 2016	HR-weighted score	LNR, lymphovascular invasion, TNM, CEA, Hb
31	30	Qu Jinglei 2015	HR-weighted score	Age, tumor size, Lauren type, T, number of positive LN, NLR
32	31	Xu Yeqiong 2016	Nomogram	Age, grade, T, N, chemotherapy, ABO blood type, CA724
33	32	Kim Jong Gwang 2008	Coefficient-weighted score	ECOG performance score, bilirubin, peritoneal metastasis, bone metastasis, number of metastasis sites
34	33	Kologlu Murat 2000	Coefficient-weighted score	T, N, M, LNR,resection margin, tumor site, lymphedenectomy, Lauren type, Borrmann type
35	34	Du Chunyan 2011	Coefficient-weighted score	Tumor size, lymphatic and/or blood vascular invasion, perineural invasion
36	35	Becker Karen 2012	Unweighted score	T, N, tumor regression

37	36	Lee J 2007	Unweighted score	ECOG performance score, bone metastasis, ascite, ALP, albumin, previous gastrectomy
38	37	Wang Jin 2016	HR-weighted score	Liver metastasis, bone metastasis, NLR, number of metatsasis sites, previous gastrectomy
39	38	Lei Ke Feng 2012	Equation	SERPINB5, KLK10, KLK11, TNM
40	39	Kadowaki Shigenori 2014	Unweighted score	ECOG performance score, multiple metastasis sites, ALP
41	40	Hirabayashi S 2014	Nomogram	Age, sex, tumor size, tumor site, macroscopic type, histological type, depth of invasion, number of positive LN, number of examined LN, lymphovascular invasion
42	41	Takahari Daisuke 2014	Unweighted score	ECOG performance score, ALP, number of metastasis sites, previous gastrectomy
43	42	Liu Hao 2015	Nomogram	T, N, M, Jagged1 expression
44	43	Song Kyo Young 2014	Nomogram	Age, sex, tumor size, T, N, Borrmann type, Lauren type
45	45	Wang Zixian 2016	Nomogram	Tumor size, T, N, FOLFOX6*THN
46	46	Liu Hao 2016(2)	Nomogram	T, N, CSF1 expression
47	47	Marubini E 1993	Coefficient-weighted score	Age, T, N, tumor site
48	48	Wang Zhiqiang 2017	Unweighted score	NR1I2, CST2, LAMP5, MMP7, LGALSL, CES1P1, COL8A1, FOXS1,C1ORF198
49	49	Wang Pin 2016	Coefficient-weighted	53 genes

			score	
50	51	Bauer L 2018	Equation	T, site, Lauren type, CCL5, CTNNB1, EXOSC3, LZTR1
51	53	Cao Yifan 2017	Nomogram	TNM, CXCR1
52	54	Cao Yifan 2016	Nomogram	TNM, gp130
53	55	Chen Hao 2017	Nomogram	Lauren type, N, metastasis, tetranectin
54	56	Chen Shangxiang 2017	Nomogram	Age, site, patholgical type, T, TLN,mLNR
55	57	Choi Jongho 2018	Equation, Nomogram	NLR, age:NLR, sex, size, T, N
56	58	De Brian 2018	Nomogram	TNM, treatment, grade, race, comorbidity score, sex
57	59	Deng Xiaorong 2017	Nomogram	Gene risk score, age, sex, size, grade, radiation
58	60	Fuchs Charles S 2017	Equation	Primary tumor, differentiation, time to progression, ECOG, peritoneal metastasis, ALP, lymphcyte, LDH, albumin, AST, neutrophil, sodium
59	61	Goshayeshi Laden 2017	Equation	Sex, birth year, age at diagnosis, family history, other GI cancer, age at diagnosis of family gastric cancer
60	63	Hou Junyi 2016	Unweighted score	TRPC1, SGCE, TNFRSF11A, LRRN1, HLF, CYS1, PPP1R14A, NOV, NBEA, CES1, RGN
61	64	Hung Yushin 2017	Unweighted score	T, LNR, CEA, ECOG performance score, adjuvant chemotherapy
62	65	Jiang Yuming 2018	Nomogram	IS, differentiation, CA199, CEA, T,N
63	65	Jiang Yuming	Nomogram	IS, differentiation, CA199, CEA, T,N

		2018		
64	67	Kim Sunyoung 2018	Nomogram	Age, ECOG performance score, differentiation, liver metastasis, lung metastasis, peritoneal metastasis, bone metastasis, gastrectomy, WBC, platelet, albumin, bilirubin, ALP
65	67	Kim Sunyoung 2018	Nomogram	Age, ECOG performance score, differentiation, liver metastasis, lung metastasis, peritoneal metastasis, bone metastasis, gastrectomy, WBC, platelet, albumin, bilirubin, ALP, response to chemotherapy
66	68	Kangi Azam Korhani 2017	Artificial Neural Network	Time of diagnosis, age, sex, smoking, addiction to opium, residency, family history, morphology, surgery, chemotherapy, radiotherapy, metastasis, histological grade, TNM
67	68	Kangi Azam Korhani 2017	Bayesian Neural Network	Time of diagnosis, age, sex, smoking, addiction to opium, residency, family history, morphology, surgery, chemotherapy, radiotherapy, metastasis, histological grade, TNM
68	69	Lin C 2017	Nomogram	T, N, Tryptase
69	70	Lin Chao 2016	Nomogram	T, N, M, IL-13Ra2
70	71	Liu Hao 2017	Nomogram	T, N, GM-CSF, adjuvant chemotherapy
71	72	Miao Yan 2017	Equation, Score	LINC01018, LOC553137, MIR4435-2HG, TTTY14
72	73	Narita Yukiya 2017	Nomogram	ECOG performance score, previous gastrectomy, HER2, ALP, LDH
73	74	Peng Peike 2016	Nomogram	T,N,M, calpain-4
74	75	Peng Pailan 2017	Coefficient-weighted score	ACOT7, CES1, IPMK, NES, PBX3, TMEM245, MIR6756, RAB11FIP4, RBPMS2, RPS27L, TPMT, TNFRSF11A

75	76	Qu Jinglei 2017	HR-weighted score	Age, T, NLR
76	77	Song Jianning 2017	Coefficient-weighted score	MiR-106, miR-15a, miR-93, miR-664
77	78	Sun Huiling 2017	Nomogram	T, N, M, surgery type, TBIL,albumin,
78	80	Wang Chenghu 2018	No information	TNM, TRIM29
79	81	Wang Pengliang 2017	Nomogram	Sex, age at operation, site, macroscopic type, LV invasion, T, number of metastasis LN, number of examined LN
80	83	Wang Zixian 2017	Nomogram	Age, year of diagnosis, race, site, size, differentiation, T, MLN, NLN
81	83	Wang Zixian 2017	Nomogram	Age, year of diagnosis, race, site, size, differentiation, T, LNR
82	83	Wang Zixian 2017	Nomogram	Age, year of diagnosis, race, site, size, differentiation, T, LODDS
83	84	Wen Ti 2017	HR-weighted score	Lauren type, TNM, TC PD-L1, IC PD-L1, IC PD-1, CD8
84	86	Wu Songyang 2016	Nomogram	T, N, galectin-8
85	87	Yang Junchi 2018	Coefficient-weighted score	10-gene score, N, TNM, mutL homolog mutation, recurrence
86	88	Yuan Shuqian 2017	Nomogram	Age, race, site, differentiation, T, LNR:ART

87	88	Yuan Shuqian 2017	Nomogram	Age, race, site, differentiation, T, LNR:ART
88	89	Zeng D 2018	Nomogram	Immunoscore, TNM, age, Lauren type
89	90	Zhang Heng 2018	No information	TNM, CD66b
90	91	Zhang Jing 2017	Nomogram	Age, TNM, chemotherapy, differentiation, tumor size, CEA
91	91	Zhang Jing 2017	Nomogram	TNM, chemotherapy, differentiation, tumor size, CEA, FPR
92	92	Zhao Linyong 2016	Nomogram	Age, tumor size, LN counts, LV invasion, T
93	93	Zheng Zhifang 2017	Coefficient-weighted score	Sacropenia, T, N,
94	94	Zhong Qing 2018	Nomogram	Sex, race, age, depth of invasion, metastasis LN, tumor size, tumor site, grade
95	95	Zhou Yixin 2018	Nomogram	T, tumor site, number of positive LN, number of negative LN
96	95	Zhou Yixin 2018	Nomogram	T, tumor site, LNR
97	95	Zhou Yixin 2018	Nomogram	T, tumor site, LODDS
98	96	Oh Sung Eun 2018	Artificial Neural Network	Age, sex, extent of resection, site, size, reseaction margin, histologic type, Lauren type, T, N, M, TNM, lymphatic invasion, venous invasion, perineural invasion, postoperative chemotherapy
99	97	Coimbra FJF 2016	Unweighted score	Age, extent of resection, chemotherapy
100	98	Hsieh Mengche 2016	Coefficient-weighted score	Peritoneal metastasis, NLR, mGPS, PG-SGA

101	99	Liu Jianjun	2016	Nomogram	Age, site, pathological type, depth of invasion, mLNR, NLR, CAR

*: The reference # is the same as those in the Online Resource 1.

Supplementary file 3. Quality assessment of model development and validation

Model #*	Reference #*	Study ID	Risk of bias: Participant selection	Predictors	Outcome	Sample size and participant flow	Analysis	Overall Judgment
**Model development**								
1	1	Hao Liu 2016	high	low	low	unclear	high	high
2	2	Wang Wei 2016	high	low	low	unclear	high	high
3	2	Wang Wei 2016	high	low	low	unclear	high	high
4	3	Rades Dirk 2015	high	low	low	high	high	high
5	4	Wang Xuefei 2012	low	low	low	unclear	low	unclear
6	4	Wang Xuefei 2012	low	low	low	unclear	low	unclear
7	5	Bria E 2013	high	low	low	unclear	low	high
8	6	Zhu Xiaodong 2015	high	low	low	high	high	high
9	7	Mohri Yasuhiko 2010	high	low	low	high	high	high
10	8	Woo Yanghee 2016	low	low	low	unclear	low	unclear
11	9	Victorzon M 1996	high	low	low	unclear	high	high
12	10	Costa Marcelo 2006	high	low	low	high	high	high
13	11	Haga Yoshio 2015	low	low	low	unclear	unclear	unclear
14	12	Catalano V 2008	high	low	low	unclear	high	high
15	13	Park Hyung Soon 2011	high	low	low	high	high	high
16	14	Wang Qing 2014	high	low	low	unclear	high	high
17	15	Kanagavel D 2010	high	low	low	unclear	high	high
18	16	Wang Xuefei 2014	high	low	low	unclear	high	high
19	17	Han Dongseok 2012	high	low	low	high	high	high
20	18	Song Bongil 2015	high	low	low	high	high	high
21	19	Xu Zhenyue 2010	high	high	low	high	high	high
22	20	Zhang Heng 2015	high	low	low	high	low	high
23	21	Wang Zhenglin 2015	high	low	low	unclear	high	high
24	22	Mohammadzadeh F 2015	high	low	low	high	high	high
25	23	Min Lingqiang 2015	high	low	low	high	high	high
26	24	Koo Dong Hoe 2011	low	low	low	unclear	high	high
27	25	Zhang H 2014	high	low	low	high	high	high
28	26	Liu Kai 2015	high	low	low	high	high	high
29	28	Eom Bang Wool 2015	high	unclear	low	high	high	high
30	29	Qian Jing 2016	high	low	low	high	low	high
31	30	Qu Jinglei 2015	high	low	low	high	high	high

32	31	Xu Yeqiong 2016	high	low	low	unclear	high	high
33	32	Kim Jong Gwang 2008	high	low	low	high	high	high
34	33	Kologlu Murat 2000	low	unclear	low	high	high	high
35	34	Du Chunyan 2011	high	low	low	high	high	high
36	35	Becker Karen 2012	high	high	low	unclear	high	high
37	36	Lee J 2007	high	low	low	high	high	high
38	37	Wang Jin 2016	high	low	low	high	high	high
39	38	Lei Ke Feng 2012	high	high	low	high	high	high
40	39	Kadowaki Shigenori 2014	high	low	low	unclear	high	high
41	40	Hirabayashi S 2014	high	low	low	high	high	high
42	41	Takahari Daisuke 2014	low	low	low	low	high	high
43	42	Liu Hao 2015	high	low	low	unclear	high	high
44	43	Song Kyo Young 2014	high	low	low	high	high	high
45	45	Wang Zixian 2016	low	low	low	unclear	high	high
46	46	Liu Hao 2016	high	low	low	unclear	high	high
47	47	Marubini E 1993	low	low	low	unclear	high	high
48	48	Wang Zhiqiang 2017	high	high	low	high	high	high
49	49	Wang Pin 2016	high	high	low	high	high	high
50	51	Bauer L 2018	high	low	low	high	high	high
51	53	Cao Yifan 2017	high	low	low	unclear	high	high
52	54	Cao Yi fan 2016	high	low	low	unclear	high	high
53	55	Chen Hao 2017	high	low	low	unclear	high	high
54	56	Chen Shangxiang 2017	high	low	low	unclear	high	high
55	57	Choi Jongho 2018	high	low	low	unclear	low	high
56	58	De Brian 2018	high	low	low	high	high	high
57	59	Deng Xiaorong 2017	high	high	low	unclear	high	high
58	60	Fuchs Charles S 2017	low	low	low	high	high	high
59	61	Goshayeshi Laden 2017	high	high	low	high	high	high
60	63	Hou Junyi 2016	high	high	low	high	high	high
61	64	Hung Yushin 2017	high	low	low	unclear	low	high
62	65	Jiang Yuming 2018	high	low	low	unclear	low	high
63	65	Jiang Yuming 2018	high	low	low	unclear	low	high
64	67	Kim Sunyoung 2018	high	low	low	unclear	high	high
65	67	Kim Sunyoung 2018	high	low	low	unclear	high	high
66	68	Kangi Azam Korhani 2017	high	low	low	unclear	high	high

67	68	Kangi Azam Korhani 2018	high	low	low	unclear	high	high
68	69	Lin C 2017	high	high	low	unclear	high	high
69	70	Lin Chao 2016	high	high	low	unclear	high	high
70	71	Liu Hao 2017	high	high	low	unclear	high	high
71	72	Miao Yan 2017	high	high	low	unclear	high	high
72	73	Narita Yukiya 2017	high	low	low	low	high	high
73	74	Peng Peike 2016	high	high	low	unclear	high	high
74	75	Peng Pailan 2017	high	high	low	high	low	high
75	76	Qu Jinglei 2017	high	low	low	unclear	high	high
76	77	Song Jianning 2017	high	high	low	high	high	high
77	78	Sun Huiling 2017	high	high	low	unclear	high	high
78	80	Wang Chenghu 2018	high	high	low	unclear	high	high
79	81	Wang Pengliang 2017	high	low	low	high	high	high
80	83	Wang Zixian 2017	high	low	low	low	high	high
81	83	Wang Zixian 2017	high	low	low	low	high	high
82	83	Wang Zixian 2017	high	low	low	low	high	high
83	84	Wen Ti 2017	high	high	low	high	high	high
84	86	Wu Songyang 2016	high	high	low	high	high	high
85	87	Yang Junchi 2018	high	high	low	high	high	high
86	88	Yuan Shuqian 2017	high	low	low	low	low	high
87	88	Yuan Shuqian 2017	high	low	low	low	low	high
88	89	Zeng D 2018	high	low	low	high	low	high
89	90	Zhang Heng 2018	high	high	low	unclear	high	high
90	91	Zhang Jing 2017	high	low	low	high	high	high
91	91	Zhang Jing 2017	high	low	low	high	high	high
92	92	Zhao Linyong 2016	high	low	low	unclear	high	high
93	93	Zheng Zhifang 2017	high	low	low	high	high	high
94	94	Zhong Qing 2018	high	low	low	unclear	high	high
95	95	Zhou Yixin 2018	high	low	low	high	low	high
96	95	Zhou Yixin 2018	high	low	low	high	low	high
97	95	Zhou Yixin 2018	high	low	low	high	low	high
98	96	Oh Sung Eun 2018	high	low	low	unclear	high	high
99	97	Coimbra FJF 2016	high	high	low	high	high	high
100	98	Hsieh Mengche 2016	high	low	low	high	high	high
101	99	Liu Jianjun 2016	high	low	low	unclear	high	high
**External validation**								
5	4	Wang Xuefei 2012	high	low	low	high	unclear	high

6	4	Wang Xuefei 2012	high	low	low	high	unclear	high
10	8	Woo Yanghee 2016	high	low	low	unclear	low	high
10	8	Woo Yanghee 2016	high	low	low	unclear	low	high
10	8	Woo Yanghee 2016	high	low	low	unclear	low	high
10	8	Woo Yanghee 2016	high	low	low	unclear	low	high
10	50	Akgul O 2018	high	low	low	high	low	high
10	85	Woo Yanghee 2017	high	low	low	low	unclear	high
13	62	Haga Yoshio 2018	high	low	low	low	high	high
19	17	Han Dongseok 2012	high	low	low	high	high	high
19	52	Bencivenge M 2017	high	low	low	low	high	high
19	52	Bencivenge M 2017	high	low	low	low	low	high
19	66	Kim Pyeongsu 2017	high	low	low	low	high	high
19	82	Wang ZX 2017	high	low	low	low	unclear	high
27	25	Zhang H 2014	high	low	low	high	high	high
29	27	Reim Daniel 2015	high	low	low	low	low	high
29	28	Eom Bang Wool 2015	high	low	low	low	unclear	high
42	79	Daisuke Takahari 2017	high	low	low	low	high	high
47	44	Mariani Luigi 2005	high	low	low	high	high	high
47	47	Marubini E 1993	low	low	low	unclear	high	high
48	48	Wang Zhiqiang 2017	high	low	low	low	high	high
54	56	Chen Shangxiang 2017	high	low	low	low	low	high
54	56	Chen Shangxiang 2017	high	low	low	low	low	high
60	63	Hou Junyi 2016	high	low	low	unclear	unclear	high
72	73	Narita Yukiya 2017	high	low	low	low	unclear	high
74	75	Peng Pailan 2017	high	low	low	unclear	unclear	high
80	83	Wang Zixian 2017	high	low	low	low	high	high
81	83	Wang Zixian 2017	high	low	low	low	high	high
82	83	Wang Zixian 2017	high	low	low	low	high	high
85	87	Yang Junchi 2018	high	low	low	unclear	unclear	high
89	90	Zhang Heng 2018	high	low	low	unclear	unclear	high
94	94	Zhong Qing 2018	high	low	low	unclear	high	high

*: The model # and reference # are the same as those in the Online Resource 1.
